# Association between short-term neurological outcomes and extreme hyperoxia in patients with out-of-hospital cardiac arrest who underwent extracorporeal cardiopulmonary resuscitation: a retrospective observational study from a multicenter registry

**DOI:** 10.1186/s12872-022-02598-6

**Published:** 2022-04-11

**Authors:** Masahiro Kashiura, Hideto Yasuda, Yuki Kishihara, Keiichiro Tominaga, Masaaki Nishihara, Ken-ichi Hiasa, Hiroyuki Tsutsui, Takashi Moriya

**Affiliations:** 1 Department of Emergency and Critical Care Medicine, Saitama Medical Center, Jichi Medical University, 1-847 Amanuma-cho, Omiya-ku, Saitama-shi, Saitama, 330-8503 Japan; 2 Faculty of Medical Sciences, Kyushu University, Fukuoka, Japan; 3 Kyushu University Hospital, Fukuoka, Japan

**Keywords:** Blood gas analysis, Cardiopulmonary resuscitation, Extracorporeal membrane oxygenation, Heart arrest, Hyperoxia, Post-cardiac arrest syndrome

## Abstract

**Background:**

To investigate the impact of hyperoxia that developed immediately after extracorporeal membrane oxygenation (ECMO)-assisted cardiopulmonary resuscitation (ECPR) on patients’ short-term neurological outcomes after out-of-hospital cardiac arrest (OHCA).

**Methods:**

This study retrospectively analyzed data from the Japanese OHCA registry from June 2014 to December 2017. We analyzed adult patients (≥ 18 years) who had undergone ECPR. Eligible patients were divided into the following three groups based on their initial partial pressure of oxygen in arterial blood (PaO_2_) levels after ECMO pump-on: normoxia group, PaO_2_ ≤ 200 mm Hg; moderate hyperoxia group, 200 mm Hg < PaO_2_ ≤ 400 mm Hg; and extreme hyperoxia group, PaO_2_ > 400 mm Hg. The primary and secondary outcomes were 30-day favorable neurological outcomes. Logistic regression statistical analysis model of 30-day favorable neurological outcomes was performed after adjusting for multiple propensity scores calculated using pre-ECPR covariates and for confounding factors post-ECPR.

**Results:**

Of the 34,754 patients with OHCA enrolled in the registry, 847 were included. The median PaO_2_ level was 300 mm Hg (interquartile range: 148–427 mm Hg). Among the eligible patients, 277, 313, and 257 were categorized as normoxic, moderately hyperoxic, and extremely hyperoxic, respectively. Moderate hyperoxia was not significantly associated with 30-day neurologically favorable outcomes compared with normoxia as a reference (adjusted odds ratio, 0.86; 95% confidence interval: 0.55–1.35; *p* = 0.51). However, extreme hyperoxia was associated with less 30-day neurologically favorable outcomes when compared with normoxia (adjusted odds ratio, 0.48; 95% confidence interval: 0.29–0.82; *p* = 0.007).

**Conclusions:**

For patients with OHCA who received ECPR, extreme hyperoxia (PaO_2_ > 400 mm Hg) was associated with 30-day poor neurological outcomes. Avoidance of extreme hyperoxia may improve neurological outcomes in patients with OHCA treated with ECPR.

**Supplementary Information:**

The online version contains supplementary material available at 10.1186/s12872-022-02598-6.

## Background

Out-of-hospital cardiac arrest (OHCA) is characterized by the loss of cardiac function and the absence of systemic circulation. The American Heart Association reports that the survival rate of patients with OHCA at hospital discharge is approximately 10%, which remains low despite advances in cardiopulmonary resuscitation and post-cardiac arrest syndrome management [1].

Extracorporeal membrane oxygenation (ECMO)-assisted cardiopulmonary resuscitation (ECPR) is the application of venoarterial extracorporeal membrane oxygenation in patients whose cardiac arrest is refractory to conventional cardiopulmonary resuscitation [2]. ECPR has been shown to improve clinical outcomes for patients with OHCA [3–5]. The main purpose of ECPR is to restore blood circulation and gas exchange. ECMO provides time for cardiopulmonary interventions that are necessary to obtain adequate spontaneous circulation, including percutaneous coronary intervention, pulmonary thrombectomy, and rewarming.

Existing literature on the potential use of ECMO reports that hyperoxia contributes to the deterioration of patients with post-cardiac arrest syndrome (PCAS) [6]. Therefore, the latest guidelines recommend avoiding hyperoxia after the return of spontaneous circulation [7, 8]. In ECPR, supraphysiological levels of oxygenation are created by the fraction of oxygen in the sweep gas (FDO_2_), which can lead to the exacerbation of PCAS. However, clinical studies evaluating hyperoxia associated with ECPR are limited [9–11]. Therefore, the influence of hyperoxia on neurological outcomes and mortality in patients with OHCA undergoing ECPR remains unclear.

This study aimed to investigate the relationship between hyperoxia and short-term neurological outcomes in adult patients who underwent ECPR. We focused on the partial pressure of oxygen in arterial blood (PaO_2_) levels in the early phases after ECMO pump-on.

## Methods

### Study design and setting

This study is reported as per the Strengthening the Reporting of Observational Studies in Epidemiology guideline (Additional file 1). The current study was a retrospective analysis of data from the Japanese Association for Acute Medicine Out-of-Hospital Cardiac Arrest (JAAM-OHCA) registry that were collected between June 2014 and December 2017. This registry provides for the nationwide, multicenter, prospectively focused collection of pre-hospital and in-hospital data from patients with OHCA in Japan [12]. The registry included all OHCA patients who were transported to participating institutions. Pre-hospital data were obtained from the All-Japan Utstein Registry of the Fire and Disaster Management Agency, as previously reported [13]. In-hospital data were collected via an internet-based system by physicians or medical staff at each institution. The JAAM-OHCA registry committee integrated pre- and in-hospital data, as previously described [14].

### Participants

In this study, we included adult patients (age ≥ 18 years) in the registry who were introduced to ECMO during cardiac arrest in the emergency room. Patients for whom no PaO_2_ data were available after ECMO initiation were excluded. In addition, we excluded patients who experienced hypoxia (initial measurement of PaO_2_ < 60 mm Hg) after the start of ECMO.

### Data collection

Patient demographics and pre-hospital factors were extracted from the JAAM-OHCA registry. The data was segmented as follows: age, sex, witness status (emergency medical service personnel or others), presence of a bystander who performed cardiopulmonary resuscitation, etiology of cardiac arrest (cardiac or non-cardiac), initial cardiac rhythm, pre-hospital adrenaline administration, pre-hospital airway management, pre-hospital shock delivery, and response time (time from call to scene arrival, time from scene to hospital arrival). In addition, in-hospital factors and outcomes were extracted as follows: cardiac rhythm on arrival, in-hospital shock delivery, in-hospital adrenaline administration, antiarrhythmic drug administration, transient return of spontaneous circulation before ECMO pump-on, time from hospital arrival to ECMO pump-on, time from hospital arrival to initial blood gas analysis after ECMO pump-on, initial blood gas analysis data (pH, PaO_2_, partial pressure of arterial carbon dioxide [PaCO_2_], bicarbonate ion concentration, lactate level) after ECMO pump-on, intra-aortic balloon pump use, percutaneous coronary intervention, targeted temperature management, and cerebral performance category 30 days after cardiac arrest.

### Exposure and definition

We divided the eligible patients into three groups according to their initial PaO_2_ levels after ECMO pump-on. The three groups were as follows: normoxia group, 60 mm Hg ≤ PaO_2_ ≤ 200 mm Hg; moderate hyperoxia group, 200 mm Hg < PaO_2_ ≤ 400 mm Hg; and extreme hyperoxia group, PaO_2_ > 400 mm Hg.

### Outcome measures

Outcomes were assessed by emergency physicians at participating hospitals 30 days after cardiac arrest. The primary outcome was a 30-day neurologically favorable outcome after cardiac arrest. A neurologically favorable outcome was defined as a cerebral performance category of 1 or 2. The cerebral performance categories included the following five outcomes: (1) good cerebral recovery, (2) moderate cerebral disability, (3) severe cerebral disability, (4) coma or vegetative state, and (5) death or brain death [15]. The secondary outcome was 30-day survival after cardiac arrest.

### Statistical analyses

Descriptive statistics were calculated for all variables of interest. Continuous variables are reported as medians and interquartile ranges (IQRs), while categorical variables are summarized using counts and percentages. Categorical variables in the three groups were analyzed using the Chi-square test, and continuous variables were analyzed using the Kruskal–Wallis test. Cubic splines were used to examine the potential nonlinear effects of PaO_2_ levels on 30-day neurologically favorable outcomes.

We used multiple imputations to compensate for missing data, and ten imputed datasets were generated [16]. Univariate logistic regression analysis was performed to calculate the crude odds ratio (OR) of the PaO_2_ level group for 30-day favorable neurological outcomes or 30-day survival after cardiac arrest.

Subsequently, we performed multiple propensity score analysis in the multivariate analysis in order to adjust and control for multiple independent variables [17]. A multiple propensity score is a conditional probability of patients being categorized into three or more groups given baseline covariates. Multiple propensity score analysis was applied to compare three or more groups [18]. First, we performed a multinomial logistic regression analysis by setting one of the three PaO_2_ groups as the dependent variable. The following covariates were used to calculate the multiple propensity scores: age, sex, witness, bystander administration of cardiopulmonary resuscitation, initial cardiac rhythm, pre-hospital shock delivery, pre-hospital adrenaline administration, pre-hospital advanced airway management, time from call to scene, time from scene to hospital arrival, etiology, cardiac rhythm on arrival, transient return of spontaneous circulation before ECMO pump-on, time from hospital arrival to ECMO pump-on, and time from hospital arrival to blood gas analysis.

Second, we performed a binomial logistic regression analysis to determine the adjusted ORs of the PaO_2_ level group for 30-day favorable neurological outcomes or 30-day survival after cardiac arrest, adjusting for multiple propensity scores and in-hospital variables, including PaCO_2_, bicarbonate ion concentration, lactate level, intra-aortic balloon pump use, percutaneous coronary intervention, and targeted temperature management.

For robustness, we performed the sensitivity analysis (multivariate logistic regression analysis for 1-month neurological favorable outcomes and 1-month survival after cardiac arrest) using data of the cases with all characteristics recorded (complete case analysis).

ORs and 95% confidence intervals (CIs) were calculated. All statistical tests were two-sided, and a *p* value of < 0.05 was considered significant. All statistical analyses were conducted using R 3.3.1 (R Foundation for Statistical Computing, Vienna, Austria) and SPSS 24.0 for Mac (IBM Corp., Armonk, NY, USA).

## Results

### Patient enrollment

**Fig. 1:**
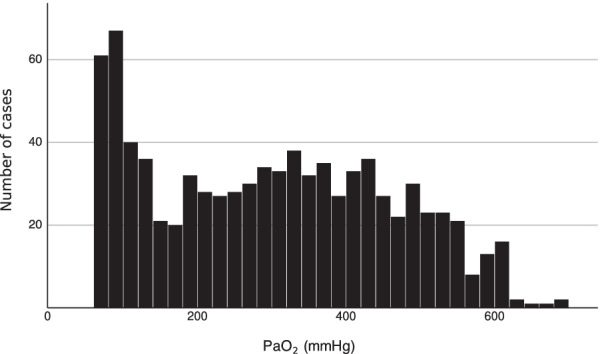
Distribution of partial pressure of arterial oxygen levels after the initiation of extracorporeal membrane oxygenation. PaO_2_, partial pressure of arterial oxygen

During the study period, 34,754 patients with OHCA were enrolled in the JAAM-OHCA registry. Of these, 1,442 patients underwent ECPR. After excluding 113 patients with PaO_2_ < 60 mm Hg and 462 for missing blood gas analysis, 847 were finally included in the study (Fig. 1).

### Patient characteristics and outcomes

The demographic, pre-hospital, and in-hospital characteristics of patients are shown in Table 1. The median age was 62 years (IQR, 49–69 years), and 79.1% of patients were male. The outcome variables are listed in Table 2. Of the total patients, 280 (33.1%) survived for 30 days after OHCA, and 140 (16.5%) had favorable 30-day neurological outcomes.

**Table 1:** Demographic, pre-hospital, and in-hospital characteristics of the study population divided into groups based on initial partial pressure of arterial oxygen levels after the start of extracorporeal membrane oxygenation

Variables	Total (n = 847)	Normoxia (n = 277)	Moderate hyperoxia (n = 313)	Extreme hyperoxia (n = 257)	*p* value
*Pre-hospital variables*					
Age (years)	62 (49–69)	58 (49–68)	62 (49–69)	64 (52–72)	0.017
Male, n (%)	670 (79.1)	223 (80.5)	258 (82.4)	189 (73.5)	0.091
Witness, n (%)					0.95
EMS personnel	115 (15.7)	38 (16.2)	42 (16.1)	35 (14.8)	
Others	464 (63.5)	148 (63.2)	162 (62.1)	154 (65.3)	
Bystander CPR, n (%)	326 (44.6)	102 (43.6)	120 (46.0)	104 (44.1)	0.74
Initial cardiac rhythm monitored, n (%)					0.55
Vf/pulseless VT	463 (63.3)	141 (60.3)	177 (67.8)	145 (61.4)	
PEA	149 (20.4)	43 (18.4)	52 (19.9)	54 (22.9)	
Asystole	66 (9.0)	29 (12.4)	18 (6.9)	19 (8.1)	
Others	53 (7.3)	21 (9.0)	14 (5.4)	18 (7.6)	
Pre-hospital shock delivery, n (%)	548 (75.0)	168 (71.8)	207 (79.3)	173 (73.3)	0.32
Pre-hospital adrenaline administration, n (%)	253 (34.6)	75 (32.1)	89 (34.1)	89 (37.7)	0.48
Pre-hospital advanced airway management, n (%)	378 (51.8)	117 (50.0)	145 (55.8)	116 (49.2)	0.51
Time from call to scene (mins)	7 (5–8)	7 (5–8)	7 (5–8)	7 (5–8)	0.86
Time from scene to hospital arrival (mins)	25 (19–32)	24 (19–31)	25 (19–34)	24 (19–30)	0.46
Cardiac origin, n (%)	715 (84.4)	226 (81.6)	266 (85.0)	223 (86.8)	0.059
*In-hospital variables*					
Cardiac rhythm on arrival, n (%)					0.003
Vf/pulseless VT	374 (44.2)	102 (36.8)	144 (46.0)	128 (49.8)	
PEA	241 (28.5)	74 (26.7)	96 (30.7)	71 (27.6)	
Asystole	164 (19.4)	69 (24.9)	51 (16.3)	44 (17.1)	
Others	68 (8.0)	32 (11.6)	22 (7.0)	14 (5.4)	
In-hospital shock delivery, n (%)	539 (63.6)	151 (54.5)	220 (70.3)	168 (65.4)	< 0.001
In-hospital adrenaline administration, n (%)	747 (88.5)	236 (85.8)	273 (87.5)	238 (92.6)	0.031
Antiarrhythmic drug administration, n (%)	406 (48.3)	110 (40.0)	159 (51.5)	137 (53.3)	0.002
Transient ROSC before ECMO pump-on, n (%)	312 (37.1)	143 (51.8)	97 (31.4)	72 (28.0)	< 0.001
Time from hospital arrival to ECMO pump-on (mins)	32 (21–47)	35 (24–61)	30 (20–46)	30 (20–40)	< 0.001
Time from hospital arrival to BGA (mins)	60.5 (29–159)	40 (18–151)	78 (39–192)	60 (35–123.5)	< 0.001
pH	7.14 (6.95–7.29)	7.07 (6.88–7.25)	7.2 (7.01–7.31)	7.16 (6.96–7.30)	< 0.001
PaO_2_ (mmHg)	300 (148–427)	106 (83–145)	311 (254–351)	489 (439–537)	< 0.001
PaCO_2_ (mmHg)	39 (31–52)	48 (36–71)	37 (31–47)	36 (29–45)	< 0.001
HCO_3_^−^ (mEq/L)	13.3 (9.7–17.2)	14.0 (10.4–17.3)	13.5 (9.9–17.5)	12.3 (8.5–15.8)	0.002
Lactate (mEq/L)	11.8 (6.9–16.0)	11.2 (6.1–15.9)	11.8 (6.6–16.0)	12.8 (8.3–16.0)	0.076
Intra-aortic balloon pump use, n (%)	531 (62.7)	180 (65.0)	191 (61.0)	160 (62.3)	0.53
Percutaneous coronary intervention, n (%)	348 (41.1)	122 (44.0)	112 (35.8)	114 (44.4)	0.004
Targeted temperature management, n (%)	469 (55.4)	161 (58.1)	165 (52.7)	143 (55.6)	0.39

Data are presented as medians (interquartile ranges) for continuous variables and numbers (proportions) for categorical values. Proportions exclude missing data

*BGA* blood gas analysis, *CPR* cardiopulmonary resuscitation, *ECMO* extracorporeal membrane oxygenation, *EMS* emergency medical service, *HCO*_*3*_^−^ bicarbonate ion, *PaCO*_*2*_ partial pressure of arterial carbon dioxide, *PaO*_*2*_ partial pressure of arterial oxygen, *PEA* pulseless electrical activity, *ROSC* return of spontaneous circulation, *Vf* ventricular fibrillation, *VT* ventricular tachycardia

**Table 2:** Outcomes of the study population divided into groups based on the initial partial pressure of arterial oxygen level after the start of extracorporeal membrane oxygenation

Outcomes	Total (n = 847)	Normoxia (n = 277)	Moderate hyperoxia (n = 313)	Extreme hyperoxia (n = 257)	*p* value
30-day favorable neurological outcome, n (%)	140 (16.5)	54 (19.5)	57 (18.2)	29 (11.3)	0.083
30-day survival, n (%)	280 (33.1)	102 (36.8)	108 (34.5)	70 (27.2)	0.10
30-day CPC, n (%)					0.068
1. Good cerebral recovery	94 (11.1)	35 (12.6)	40 (12.8)	19 (7.4)	
2. Moderate cerebral disability	46 (5.4)	19 (6.9)	17 (5.4)	10 (3.9)	
3. Severe cerebral disability	60 (7.1)	13 (4.7)	27 (8.6)	20 (7.8)	
4. Coma or vegetative state	80 (9.4)	35 (12.6)	24 (7.7)	21 (8.2)	
5. Death or brain death	567 (66.9)	175 (63.2)	205 (65.5)	187 (72.8)	

Data are presented as numbers (proportions)

*CPC* cerebral performance category, *ECMO* extracorporeal membrane oxygenation, *PaO*_*2*_ partial pressure of arterial oxygen

### PaO_2_ distribution, grouping, and nonlinear association with neurological outcomes

The median PaO_2_ level was 300 mm Hg (IQR, 148–427 mm Hg), and the distribution of PaO_2_ levels is shown in Fig. 1. A total of 277, 313, and 257 patients were categorized as normoxic, moderately hyperoxic, and extremely hyperoxic, respectively.

**Fig. 2:**
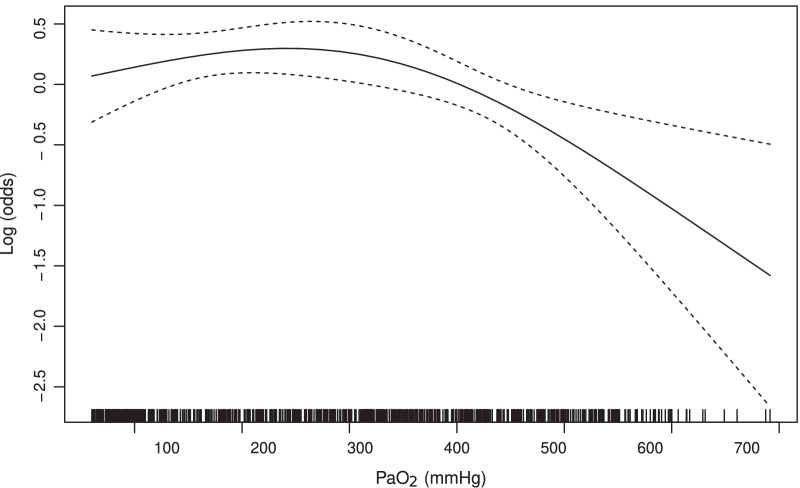
Nonlinear relationship between the logarithm of odds for 30-day favorable neurological outcome according to partial pressure of arterial oxygen levels. The solid line indicates the cubic spline curve of the logarithm of odds. Dotted lines indicate the 95% confidence interval. PaO_2_, partial pressure of arterial oxygen

The cubic spline curve demonstrated a nonlinear association between PaO_2_ levels and log (odds). This means that the odds of a favorable neurological outcome were > 1.0 when log (odds) was > 0 (Fig. 2).

### Logistic regression analysis

The results of univariate and multivariate logistic regression analyses for neurological outcome at 30 days and survival at 30 days are shown in Table 3. Moderate hyperoxia was not significantly associated with neurological outcome compared with normoxia as a reference (crude OR, 0.92; 95% CI 0.61–1.39; *p* = 0.69), while extreme hyperoxia was significantly associated with deterioration in neurological outcome compared with normoxia (crude OR, 0.53; 95% CI 0.32–0.86; *p* = 0.010).

**Table 3:** Univariate and multivariate logistic regression analyses of 30-day favorable neurological outcomes and 30-day survival after cardiac arrest

	Crude OR (95% CI)	*p* value	Adjusted OR (95% CI)	*p* value
*For 30-day favorable neurological outcomes*				
Normoxia	Reference		Reference	
Moderate hyperoxia	0.92 (0.61–1.39)	0.69	0.86 (0.55–1.35)	0.51
Extreme hyperoxia	0.53 (0.32–0.86)	0.010	0.48 (0.29–0.82)	0.007
*For 30-day survival*				
Normoxia	Reference		Reference	
Moderate hyperoxia	0.90 (0.65–1.27)	0.56	0.92 (0.63–1.34)	0.66
Extreme hyperoxia	0.64 (0.45–0.93)	0.018	0.66 (0.44–1.00)	0.048

Multiple propensity scores were calculated by adjusting for the following factors: age, sex, witness, bystander cardiopulmonary resuscitation, initial cardiac rhythm, pre-hospital shock delivery, pre-hospital adrenaline administration, pre-hospital advanced airway management, time from call to scene, time from scene to hospital arrival, etiology, cardiac rhythm on arrival, transient return of spontaneous circulation before ECMO pump-on, time from hospital arrival to ECMO pump-on, and time from hospital arrival to blood gas analysis. Adjusted ORs were calculated, controlling for the multiple propensity scores and the following variables: PaCO_2_, HCO_3_^−^ concentration, lactate level, intra-aortic balloon pump use, percutaneous coronary intervention, and targeted temperature management

*CI* confidence interval, *ECMO* extracorporeal membrane oxygenation, *HCO*_*3*_^−^ bicarbonate ion, *OR* odds ratio, *PaCO*_*2*_ partial pressure of arterial carbon dioxide, *PaO*_*2*_partial pressure of arterial oxygen

Even after adjusting for the covariates after ECMO and the multiple propensity scores calculated from factors before ECMO, moderate hyperoxia was not significantly associated with poor neurological outcome compared with normoxia as a reference (adjusted OR, 0.86; 95% CI 0.55–1.35; *p* = 0.51). Contrariwise, extreme hyperoxia was significantly associated with less neurologically favorable outcomes compared with normoxia (adjusted OR, 0.48; 95% CI 0.29–0.82; *p* = 0.007).

Univariate and multivariate analyses of 30-day survival also showed that extreme hyperoxia was significantly associated with mortality compared with normoxia (adjusted OR, 0.66; 95% CI: 0.44–1.00; *p* = 0.048).

### Complete case analysis as a sensitivity analysis

In the complete case analysis, a total of 663 cases were analyzed, after 184 cases were excluded due to missing data. Moderate hyperoxia was not significantly associated with neurological outcome compared with normoxia as a reference (adjusted OR, 0.92; 95% CI 0.53–1.58; *p* = 0.75) (Additional file 2: Table S1). Extreme hyperoxia was also not significantly associated with neurologically favorable outcomes compared with normoxia (adjusted OR, 0.64; 95% CI 0.36–1.13; *p* = 0.13).

## Discussion

### Main findings

We investigated the relationship between hyperoxia and short-term neurological outcomes in adult patients who underwent ECPR, focusing on PaO_2_ levels in the early phases after ECMO. Two-thirds of patients undergoing ECPR were exposed to moderate or greater hyperoxia (PaO_2_ > 200 mm Hg). There was a nonlinear relationship between PaO_2_ levels in the early phase after ECMO initiation and neurological outcome 30 days after cardiac arrest in patients with OHCA who underwent ECPR. Extreme hyperoxia (PaO_2_ > 400 mm Hg) was significantly associated with worse neurological outcomes and higher mortality at 30 days after adjusting for multiple confounders.

Sensitivity analysis showed that hyperoxia tended to be associated with a worse neurological prognosis; however, it could not demonstrate significance. This might have been due to the reduced power, as 22% of the cases were excluded due to missing values.

### Effect of hyperoxia in patients with cardiac arrest

Dissolved oxygen that accumulates in the arteries is thought to have adverse effects through a complex combination of mechanisms, including excessive production of reactive oxygen species, pulmonary toxicity, and cardiac and neurological effects [19]. The toxicity of reactive oxygen species is the result of lipid peroxidation, protein oxidation, and DNA damage. When lipid peroxidation affects intracellular or extracellular membranes, it causes enzyme inactivation, thiol oxidation, and inhibition of the mitochondrial respiratory chain [20]. The oxidation of proteins results in resistance to proteolysis by aggregation [21]. The toxic effect of reactive oxygen species on DNA is dominated by cell cycle alterations, apoptosis, and carcinogenesis [22].

Animal studies have shown adverse effects of hyperoxia after cardiac arrest, including increased cell death by apoptosis and cytokine production [23, 24]. Many observational studies and meta-analyses have shown that hyperoxia after cardiac arrest is associated with poor neurological outcomes and increased mortality in post-cardiac arrest patients [25–28]. However, several controversial studies have reported no association between hyperoxia and clinical outcomes [23, 29, 30]. Two randomized controlled trials are currently ongoing to determine the appropriate oxygenation threshold in post-cardiac arrest patients (NCT03138005 and NCT03141099) [31, 32].

### Effect of hyperoxia in patients undergoing ECPR

Oxygenation at supraphysiological levels is common during venoarterial ECMO, including ECPR, which may result in extreme hyperoxia, depending on the FDO_2_ setting of the sweep gas [11]. Therefore, cardiac arrest patients undergoing ECPR are at higher risk of hyperoxia than those only receiving conventional CPR. Two-thirds of patients undergoing ECPR in this study were exposed to moderate or greater hyperoxia (Fig. 1).

Previous single-center and multicenter studies have reported a relationship between exposure to hyperoxia and poor clinical outcomes (mortality and impaired neurological status) in patients with cardiac arrest undergoing ECPR [9–11, 33, 34]. The results of these studies are consistent with those of the present study. However, confounding factors were not adjusted and controlled for. This was due to the small number of patients in the previous studies.

### Clinical applications and strength of the present study

In this study, we focused on blood gas levels during the initial period after the start of ECMO. Avoiding early extreme hyperoxia may improve outcomes in patients undergoing ECPR. Therefore, it may be necessary to adjust the FDO_2_ of the ECMO sweep gas immediately after the ECMO pump is turned on, to maximize the effect of ECPR on improving clinical outcomes.

Our study had several strengths. First, it included a larger sample size than that available in previous studies that assessed hyperoxia in ECPR (9–11, 32). Second, with the benefit of a large sample size and multiple propensity scores, we were able to evaluate the impact of hyperoxia on clinical outcomes after controlling for various confounders. Third, the effect of immortal bias was minimal because we evaluated blood gas data early after the start of ECMO (median time, 1 h after patient presentation).

### Study limitations

This study had several limitations. First, the ECPR protocols in each participating institution were standardized. Differences in practices among the facilities may have skewed the results, even after adjusting for various confounders. Second, several clinical outcomes, including ECMO duration and length of hospital and intensive care unit stay, were not registered in the JAAM-OHCA registry. Therefore, the effect of hyperoxia on these outcomes remains unclear. Third, two-thirds of patients who underwent ECPR were excluded because of missing blood gas data in the present study. The missing data may have distorted the results even though our study was strengthened by a large sample size. Fourth, we did not have data regarding cardiac function. During ECPR, peripheral venoarterial ECMO is usually used [35]. A watershed zone known as Harlequin syndrome (North–South syndrome) develops because the retrograde flow from ECMO mixes with the antegrade blood flow from the patient’s own heart [36]. To avoid the influence of the North–South syndrome, we excluded patients with hypoxia. However, PaO_2_ may be affected by the patient’s cardiac function. Similarly, details of cardiac treatment, such as percutaneous coronary intervention, have not been evaluated. Fifth, we chose PaO_2_ cutoff values of 200 mm Hg and 400 mm Hg in this study. The cutoff value of 200 mm Hg was chosen because it had been used in previous studies. However, the 400 mm Hg cutoff value was arbitrarily selected before analysis [9, 34] and may not have been appropriate. Furthermore, we have not been able to evaluate the exposure duration of hyperoxia. In view of the limitations of this study, future well-designed studies are required.

## Conclusions

Extreme hyperoxia immediately after ECMO was associated with deterioration of short-term neurological outcomes and survival in patients with OHCA who underwent ECPR. Avoidance of extreme hyperoxia may improve neurological outcomes in patients with OHCA treated with ECPR. Further studies are required to determine the optimal PaO_2_ treatment level.

## Supplementary Information

**Additional file 1:** STROBE statement checklist.

**Additional file 2:** Complete case analysis as a sensitivity analysis.

**Additional file 3:** The list of participating hospitals and approval numbers for the Institutional Review Board of each institution.

## Data Availability

The use of JAAM-OHCA registry data is limited to members of the Japanese Assciation of Acute Medicine, and permission must be obtained from the society [12].

## Abbreviations

CI: Confidence interval
ECMO: Extracorporeal membrane oxygenation
ECPR: Extracorporeal membrane oxygenation-assisted cardiopulmonary resuscitation
FDO_2_: Fraction of oxygen in the sweep gas
JAAM: Japanese Association for Acute Medicine
OHCA: Out-of-hospital cardiac arrest
OR: Odds ratio
PaO_2_: Partial pressure of oxygen in arterial blood
PCAS: Post-Cardiac Arrest Syndrome
