# Supplementary material for: Association between short-term neurological outcomes and extreme hyperoxia in patients with out-of-hospital cardiac arrest who underwent extracorporeal cardiopulmonary resuscitation: a retrospective observational study from a multicenter registry
Source: BMC Cardiovasc Disord. 2022 Apr 11;22:163. doi: 10.1186/s12872-022-02598-6 (PMC9003952; doi:10.1186/s12872-022-02598-6)
Supplement: Supplementary file 2 — Additional file 2: Complete case analysis as a sensitivity analysis. [file 12872_2022_2598_MOESM2_ESM.docx]

**Additional file 2.**

**Table S1.** Univariate and multivariate logistic regression analyses of 30-day favorable neurological outcomes and 30-day survival after cardiac arrest in complete cases without missing data

|  | **Crude OR (95% CI)** | ***p*-value** | **Adjusted OR (95% CI)** | ***p*-value** |
| --- | --- | --- | --- | --- |
| **For 30-day favorable neurological outcomes** | |  |  |  |
| Normoxia | Reference |  | Reference |  |
| Moderate hyperoxia | 0.95 (0.57–1.57) | 0.69 | 0.92 (0.53–1.58) | 0.75 |
| Extreme hyperoxia | 0.71 (0.42–1.18) | 0.71 | 0.64 (0.36–1.13) | 0.13 |
| **For 30-day survival** |  |  |  |  |
| Normoxia | Reference |  | Reference |  |
| Moderate hyperoxia | 0.76 (0.50–1.14) | 0.18 | 0.73 (0.46–1.15) | 0.17 |
| Extreme hyperoxia | 0.76 (0.51–1.13) | 0.18 | 0.72 (0.46–1.13) | 0.15 |

Abbreviations: CI, confidence interval; ECMO, extracorporeal membrane oxygenation; HCO_3_^−^, bicarbonate ion; OR, odds ratio; PaCO_2_, partial pressure of arterial carbon dioxide; PaO_2_, partial pressure of arterial oxygen.
