# Supplementary material for: Association between short-term neurological outcomes and extreme hyperoxia in patients with out-of-hospital cardiac arrest who underwent extracorporeal cardiopulmonary resuscitation: a retrospective observational study from a multicenter registry
Source: BMC Cardiovasc Disord. 2022 Apr 11;22:163. doi: 10.1186/s12872-022-02598-6 (PMC9003952; doi:10.1186/s12872-022-02598-6)
Supplement: Supplementary file 3 — Additional file 3: The list of participating hospitals and approval numbers for the Institutional Review Board of each institution. [file 12872_2022_2598_MOESM3_ESM.docx]

**Additional file 3.** The list of participating hospitals and approval numbers for Institutional Review Board of each institution.

| **Institution** | **IRB number** |
| --- | --- |
| Osaka University Hospital | Not applicable |
| Tane General Hospital | 2020-12 |
| Kindai University Hospital | 24-062 |
| Osaka Mishima Emergency Medical Center | 2012-01A |
| Senshu Trauma and Critical Care Center | 29-4 |
| Osaka General Medical Center | 24-2020-1 |
| Osaka Police Hospital | Not applicable |
| Osaka Prefectural Nakakawachi Emergency and Critical Care Center | 02-0634-A |
| Osaka City University Hospital | 4238 |
| National Hospital Organization Osaka National Hospital | 12028 |
| Kansai Medical University Hospital | 2017042 |
| Saieikai Senri Hospital | 290504 |
| Osaka City General Hospital | 1207023 |
| Gifu University Hospital | 25-241 |
| Kyoto University Hospital | R1045 |
| Hokkaido University Hospital | Not applicable |
| Tohoku University Hospital | 2018-1-100 |
| Nihon University Itabashi Hospital | Not applicable |
| Kansai Medical University Medicine Center | 2017042 |
| Yamaguchi University Hospital | H26-5-3 |
| Kyushu University Hospital | 2020-481 |
| Akita University Hospital | 1085 |
| Niigata University Medical & Dental Hospital | 2019-0427 |
| Sapporo Medical University Hospital | 262-7 |
| Mizushima Central Hospital | 11 |
| Japan Community Health care Organization Shiga Hospital | Not applicable |
| Tokyo Bay Urayasu Ichikawa Medical Center | 79 |
| Hakodate Municipal Hospital | Not applicable |
| Araki Neurosurgical Hospital | 041 |
| Hokkaido Medical Center | 26-6-5 |
| Kakogawa Central City Hospital | 26-13 |
| Saitama Red Cross Hospital | 20140709-1 |
| Fujisawa City Hospital | F2014009 |
| Nihon University Hospital | 140702 |
| Yamanashi Prefectural Central Hospital | Not applicable |
| Yamagata Prefectural Central Hospital | 70 |
| St.Luke's International Hospital | 14-R048 |
| Ibaraki Prefectural Central Hospital | 26-34 |
| Kumamoto University Hospital | 847 |
| Takarazuka Hospital | 2019-04 |
| Iwate Prefectural Central Hospital | 1130 |
| Saga University Hospital | 2020-01-R-09 |
| Center Hospital of the National Center for Global Health and Medicine | 1649 |
| Hachinohe City Hospital | Not applicable |
| National Hospital Organization Kumamoto Medical Center | 515 |
| Nippon Medical School Musashi Kosugi Hospital | 259-26-04 |
| Nihonkai General Hospital | 25-3-2 |
| Niigata Prefectural Shibata Hospital | 121 |
| Hospital of the University of Occupational and Environmental Health | H26-157 |
| National Center for Child Health and Development | 837 |
| Iwate Medical University Hospital | H25-91 |
| National Cerebral and Cardiovascular Center | R20046 |
| Tokyo Metropolitan Children's Medical Center | H26-70 |
| Fukuyama City Hospital | 207 |
| Kyoto City Hospital | 277 |
| Nippon Medical School Tama Nagayama Hospital | 403 |
| Kagawa University Hospital | H26-122 |
| Seirei Hamamatsu General Hospital | 1783 |
| Gifu Prefectural General Medical Center | 188 |
| Nagasaki University Hospital | 15012699-4 |
| Gunma University Hospital | 14-56 |
| Yokohama City University Medical Center | D1502003 |
| Showa University Hospital | 1729 |
| Kin-ikyo Chuo Hospital | Not applicable |
| Kobe City Medical Center General Hospital | ｋ201235 |
| Shinshu University Hospital | 3124 |
| Mito Medical Center | Not applicable |
| Chiba University Hospital | 1961 |
| Okayama University Hospital | 1047 |
| Teine Keijinkai Hospital | 2018-057 |
| Yokohama City Minato Red Cross Hospital | 2015-16 |
| Tokyo Medical And Dental University Medical Hospital | M2000-2099-03 |
| Ehime University Hospital | 1507011 |
| The University of Tokyo Hospital | 10866-(3) |
| Kimitsu Chuo Hospital | 284 |
| Okinawa Chubu Hospital | H27-55 |
| Saitama Medical Center, Saitama Medical University | 1186 |
| Shin-yurigaoka General Hospital | Not applicable |
| Fujieda Municipal General Hospital | 11 |
| Miyazaki Prefectural Nobeoka Hospital | Not applicable |
| University Hospital, Kyoto Prefectural University of Medicine | ERB-C-650 |
| Maebashi Red Cross Hospital | 28-43 |
| Osaka Red Cross Hospital | 768 |
| National Hospital Organization Takasaki General Medical Center | H29-30 |
| Kishiwada Tokushukai Hospital | Not applicable |
| National Hospital Organization Nagasaki Medical Center | 29048 |
| Tohoku Medical and Pharmaceutical University Hospital | 2017-2-055 |
| Japanese Red Cross Kyoto Daini Hospital | S29-32 |
| Hyogo Prefectural Kobe Children’s Hospital | 29-88 |
| Hamamatsu University Hospital | 18-031 |
| Hyogo Emergency Medical Center | 2018002 |
| Asahikawa Medical University Hospital | 18100 |
| Nagoya University Hospital | 2018-0083 |
| University of Tsukuba Hospital | H30-22 |
| Hyogo Prefectural Awaji Medical Center | 30-55 |
| Sapporo Higashi Tokushukai Hospital | Not applicable |
| Jichi Medical University Saitama Medical Center | S19-016 |
| University of the Ryukyus Hospital | 1620 |
| Juntendo University Urayasu Hospital | 2-029 |
| Nara Prefecture General Medical Center | 532 |
| Kagoshima University Hospital | 200213 |
| Chiba Kaihin Municipal Hospital | 2021-08 |
